# Coffee Drinking and Associated Factors in an Elderly Population in Spain

**DOI:** 10.3390/ijerph15081661

**Published:** 2018-08-06

**Authors:** Laura Torres-Collado, Manuela García-de la Hera, Eva Maria Navarrete-Muñoz, Laura Maria Compañ-Gabucio, Sandra Gonzalez-Palacios, Jesús Vioque

**Affiliations:** 1 Department of Public Health, History of Medicine and Gynecology, University Miguel Hernández, Alicante Institute for Health and Biomedical Research, ISABIAL–FISABIO Foundation, 03550 Alicante, Spain; l.torres@umh.es (L.T.-C.); manoli@umh.es (M.G.-d.l.H.); enavarrete@umh.es (E.M.N.-M.); lcompan@umh.es (L.M.C.-G.); sandra.gonzalezp@umh.es (S.G.-P.); 2 Spanish Consortium for Research on Epidemiology and Public Health (CIBERESP), 28029 Madrid, Spain

**Keywords:** coffee, caffeinated, decaffeinated, elderly, consumption, factors

## Abstract

Coffee consumption is highly prevalent worldwide, and many studies have reported positive and inverse associations of coffee with many diseases. However, factors associated with coffee consumption remain poorly characterized in some populations, such as the elderly. This study aimed to assess the factors associated with total, caffeinated and decaffeinated coffee consumption in an elderly population in Spain. Data were analyzed from 903 participants, aged 65 years and above, from two population-based studies carried out in the Valencia region in Spain (Valencia Nutritional Survey (VNS) and European Eye Study (EUREYE-Study). Total, caffeinated and decaffeinated coffee consumption was assessed through two specific questions using a validated food frequency questionnaire. Information on personal characteristics, anthropometry and lifestyles was collected in personal interviews. Multinomial logistic regression analysis was used to estimate the adjusted relative risk ratios (RRR) and confidence intervals (95% CI). The prevalence of total, caffeinated and decaffeinated coffee consumption was 70%, 38% and 32%, respectively. The consumption of caffeinated coffee was positively associated with: Educational level, RRR = 1.63 (1.09–2.44); body mass index (≥30), RRR = 2.03 (1.05–3.95); tobacco smoking, RRR = 1.96 (1.13–3.39); alcohol intake [≥12 g/day category intake vs. no-alcohol intake, RRR = 6.25 (3.56–10.95)]; and energy intake (*p* < 0.05). Consumption of caffeinated coffee was negatively associated with: Age (≥75 years), RRR = 0.64 (0.43–0.94); and pre-existing hypertension, RRR = 0.67 (0.45–0.98). The consumption of decaffeinated coffee was positively associated with: Alcohol intake, RRR = 2.63 (1.19–4.64); pre-existing diabetes, RRR = 1.67 (1.06–2.62); and energy intake (*p* < 0.01). The consumption of coffee is high among elderly people in Spain. It is a novelty in this study with elderly population that tobacco smoking and alcohol drinking were the two main factors associated with higher coffee consumption. Self-reported hypertension was associated with a lower consumption of caffeinated coffee, and pre-existing diabetes was associated with a higher consumption of decaffeinated coffee. These associations should be taken into account when the health effects of coffee consumption are investigated.

## 1. Introduction

During the last few decades, coffee has received a great deal of attention due to the high global prevalence of coffee consumption and because it may affect human health, either positively or negatively. Some safety concerns are related to its caffeine content, a physiological stimulant with side effects that may affect cardiovascular outcomes [1]. However, a recent meta-analysis showed that moderate coffee consumption was inversely associated with CVD risk, with the lowest CVD risk at three to five cups per day, and that heavy coffee consumption was not associated with elevated CVD risk [2].

On the other hand, coffee is rich in bioactive substances, such as phenolic compounds and minerals with a wide range of antioxidant and anti-inflammatory effects [3,4], which can improve insulin resistance and glucose metabolism [3]. Many epidemiological studies have shown that coffee consumption decreases the incidence of major causes of death such as heart disease, type 2 diabetes and several types of cancer [3,4,5,6]. Coffee consumption also decreases the risk of neurological and mental illnesses, such as Parkinson’s and Alzheimer’s diseases [4,7]. A growing body of evidence also suggests that usual coffee consumption could reduce the incidence of chronic diseases and mortality due to the strong antioxidant properties of coffee components [4,8].

Some studies, carried out mainly with university students and other young populations [9,10,11], have reported that caffeine intake was higher in men, older people and in smokers [10,11]. However, the factors associated with the habit of coffee consumption have scarcely been investigated. To the best of our knowledge, there has been no research focused on the factors associated with coffee consumption in the general or elderly populations. Thus, the aim of this study was to assess the factors associated with total, caffeinated and decaffeinated coffee consumption in elderly population 65 years and older in Spain.

## 2. Materials and Methods

### 2.1. Study Population and Participants

We analyzed the data of 903 participants (511 women and 392 men), aged 65 years and above, who participated in two population-based surveys in the Valencia Region (Spain): The EUREYE-Spain study and the Valencia Nutrition Survey (VNS). Details of both studies have been published elsewhere [12,13,14]. The EUREYE study was a multi-center, cross-sectional study based on representative samples of elderly people aged 65 years and above, from seven European countries in 2000–2001. The study enrolled 597 subjects in the province of Alicante in the Valencia Region, Spain. The VNS was a nutritional survey that was also based on a representative sample of an adult population in the three provinces of the Valencia Region. It included 306 participants, aged 65 years and above, in 1994. Participants were interviewed at baseline by trained fieldworkers who used structured questionnaires that asked for socio-demographic details, smoking history, alcohol intake, a brief medical history, educational level, diet and lifestyle factors. Written informed consent was provided by all participants, and ethical approval for the studies was given by the Local Ethical Committee of the Hospital de San Juan and the University Miguel Hernandez, Alicante, Spain (projects FIS 00/0985 and V FP-EU, QLK6-CT-1999-02094).

### 2.2. Dietary Assessment

Participants were asked about their usual dietary intake using a semi-quantitative food frequency questionnaire (FFQ), which had a similar structure to the Willett FFQ [15] that was adapted and validated for an adult population in Spain [16,17]. The FFQ used in both studies showed a satisfactory, one-year reproducibility and validity when their nutrient and food intake estimates were compared with those from four one-week dietary records in an adult population in Valencia (17). The average of validity and reproducibility correlation coefficients for nutrient intakes (adjusted for energy intake) were 0.47 and 0.40, respectively. Regarding coffee consumption, the FFQ showed a good reproducibility after a one-year period; the correlation coefficient was R = 0.60. It has also been suggested that a single assessment done with FFQ could be adequate to evaluate usual coffee drinking habits in the medium to long term [6]. Participants reported how often, on average, they had consumed each food item during the previous year. Responses ranged from “never or less than once per month” to “six or more per day”. Serving sizes of caffeinated and decaffeinated coffee were specified in the FFQ with two items: One item for caffeinated coffee and another for decaffeinated coffee. Total coffee consumption was calculated as the sum of caffeinated and decaffeinated coffee consumption in milliliters. A cup of coffee was defined using typical sizes (about 50 mL for expresso cup or 125–150 mL for instant/brewed/ground coffee). Participants were classified in two categories, according to consumption of total, caffeinated and decaffeinated consumption. These categories were non-drinkers and ≥1 cup/daily. Nutrient intakes were obtained from food composition tables from the US Department of Agriculture [18] and other Spanish published sources [19].

### 2.3. Covariates

Information on sociodemographic variables was collected from all participants at baseline. These variables included: Sex (men; women); age (in years); study (EUREYE; VNS); educational level (<primary; ≥primary); body mass index (BMI), measured as weight in kilograms divided by the square of measured height in meters (<25 kg/m^2^, 25–30 kg/m^2^, ≥30 kg/m^2^); waist circumference (normal: 78–94 cm in men and 64–80 cm in women; moderate: 94–102 cm in men and 80–88 cm in women; and large: >102 cm in men and >88 cm in women); smoking (never, ex–smoker or current smoker); alcohol consumption (0 g/day, <12 g/day or ≥12 g/day); fruit and vegetable intake in servings (tertiles); usual physical activity at work (low, mostly at sitting position; moderate-high); physical activity at leisure–time (low, mostly at sitting position; moderate-high); sleeping time (hours/day); daily energy intake (Kcals/day); and pre-existing chronic disease at baseline (self-reported diabetes, high blood cholesterol and hypertension). In elderly populations, a high level of agreement was observed between self–reported diseases and those documented in medical records [20,21].

### 2.4. Statistical Analysis

Data were analyzed using R-project software, version 3.3.2 (R Foundation for Statistical Computing, Vienna, Austria). The applied statistical tests were bilateral, and significance was established at 0.05. Descriptive analysis was performed by using chi-square and ANOVA tests to compare the characteristics of non–drinkers and drinkers for each type of coffee. Multinomial logistic regression was used to explore the association between the independent variables, as described above, and the dependent variable of coffee consumption: non-drinkers (reference category), caffeinated coffee and decaffeinated coffee drinkers. The existence of heterogeneity was explored between the two studies and was included in the analysis using I2 and Cochran’s test [22]. As the results between studies did not show heterogeneity, the results were combined with multinomial logistic regression, adjusting for the study dichotomous variable (VNS/EUREYE study).

## 3. Results

Table 1 shows the main characteristics of participants according to coffee consumption, non-drinkers and caffeinated and decaffeinated coffee drinkers. Overall, 630 participants reported drinking some amount of coffee (69.8%).Of these, 340 reported drinking caffeinated coffee (37.7%), and 290 reported drinking decaffeinated coffee (32.1%). Caffeinated coffee was more frequently consumed by: Men; participants that were 65–74 years old; participants with higher educational level; participants with higher waist circumferences; current/past smokers; alcohol drinkers; participants that were more physically active; and participants with higher energy intakes. It was less frequently consumed among diabetics. Decaffeinated coffee was also more frequently consumed by: Alcohol drinkers; participants with self–reported diabetes and hypertension; and participants with higher energy intakes.

Table 2 shows the results of the multinomial logistic regression analysis for caffeinated and decaffeinated coffee consumption as compared to non-coffee drinkers (reference). Caffeinated coffee consumption was positively associated with: A higher educational level, RRR = 1.63 (95% CI: 1.09–2.44); a BMI ≥ 30 kg/m^2^, RRR = 2.03 (95% CI: 1.05–3.95); tobacco smoking, RRR = 1.96 (95% CI: 1.13–3.39); alcohol consumption, <12.0 g/day RRR = 2.86 (1.87–4.36) and >12 g/day RRR = 6.25 (95% CI: 3.56–10.95); and energy intake (*p* < 0.001). By contrast, older age and self–reported hypertension were associated with a lower consumption of caffeinated coffee with RRR = 0.64 (95% CI: 0.43–0.94) and RRR = 0.67 (95% CI: 0.45–0.98), respectively (Table 2). Factors associated with a higher decaffeinated coffee consumption were: Alcohol consumption, >12 g/day RRR = 2.63 (95% CI: 1.49–4.64); self-reported diabetes, RRR = 1.67 (95% CI: 1.06–2.62); and energy intake, RRR = 1.06 (95% CI: 1.01–1.10).

## 4. Discussion

This study showed that coffee consumption is highly prevalent in elderly people in Spain, and that consumption is associated with several lifestyles and health statuses in different ways for caffeinated and decaffeinated coffee consumption, respectively. Consumption of caffeinated coffee was positively associated with higher educational level, higher BMI, tobacco smoking, alcohol drinking, and a higher energy intake. It was negatively associated with self-reported hypertension and older age. Decaffeinated coffee was positively associated with alcohol drinkers, higher energy intake and self-reported diabetes.

The factors associated with coffee consumption in elderly population have scarcely been investigated. The positive association that was found, particularly with smoking, alcohol consumption and energy intake, has been partially reported in previous studies, where participants, who smoke more cigarettes and had greater alcohol consumption, had a higher caffeine intake from coffee beverages [11,23]. The association with smoking and alcohol use was stronger for caffeinated coffee consumption and weaker for decaffeinated coffee, for which the association with smoking was not significant. In accordance with previous studies, it was found that older people consumed less caffeinated coffee [24], which may be related to the higher prevalence of some chronic diseases, such as hypertension, and the clinical recommendations to decrease or even quit coffee consumption at older ages [25]. In fact, we found that hypertension was associated with a significantly lower consumption of caffeinated coffee and with a non-statistically significant higher consumption of decaffeinated coffee, which is consistent with results from other studies [25]. An inverse association between coffee consumption and hypertension has also been reported in other studies [24,26].

The positive association between diabetes and coffee consumption (particularly with decaffeinated coffee) was not consistent with the correlation found in other studies with a diabetic population that consumed less total and caffeinated coffee [25]. Current evidence suggests an inverse association between coffee consumption and the risk of some chronic diseases, such as hypertension [2,5,24,27,28] or even metabolic syndrome [29]. However, because of the traditional clinical recommendations to decrease coffee consumption in this type of patient, a reverse causation cannot be ruled out [25]. Thus, more studies are required to confirm these associations in an elderly population to explore the health effects of coffee consumption and to make any recommendations on its use.

One limitation of this related to the cross-sectional study design of the two studies, which made it difficult to establish any causality. However, coffee consumption is a habit that rarely changes over time, unless medical conditions are present. Furthermore, previous studies suggested that a single assessment could be sufficient to collect information on long-term, habitual coffee consumption [6,11]. In addition, the non-response in cross-sectional studies may be of concern if non-participants have unhealthier lifestyles (e.g., higher prevalence of smoking and drinking) because of the response bias if participants have an interest to estimate prevalence. However, coffee consumption was not very influential in the participation rate in the studies; the rate of coffee consumption in the participants was similar to that found in another study carried out in the Valencia Region with an elderly population [25].

However, the current study also presented a number of strengths. To the best of the authors’ knowledge, this study was the first to evaluate the factors associated with each type of coffee consumption in a Spanish elderly population. The study used well-defined populations, from two previous studies aimed to explore other associations following good-quality protocols and validated questionnaires [12,14,16]. Although our findings could be generalized further to the elderly population, they should be confirmed in future cohort studies in elderly populations.

## 5. Conclusions

In conclusion, this study showed that coffee consumption is highly prevalent among elderly people in Spain. Smoking and alcohol habits were the two main factors associated with a higher coffee consumption, particularly for caffeinated coffee. Self-reported hypertension was associated with a lower consumption of caffeinated coffee, whereas self-reported diabetes was associated with a higher decaffeinated coffee consumption. These associations should be taken into account when the health effects of coffee consumption are investigated.

## Figures and Tables

**Table 1:** Baseline characteristics of the 903 participants, aged 65 years and above, from the EUREYE-Spain Study and the Valencia Nutrition Survey in Spain, according to the type of coffee consumption.

	No Coffee Consumption	Caffeinated Coffee	Decaffeinated Coffee	
	No	Yes	Yes	*p* ^1^
N (%)	273 (30.2)	340 (37.7)	290 (32.1)	
Sex, n (%)				
Men	90 (23.0)	185 (47.2)	117 (29.8)	
Women	183 (35.8)	155 (30.3)	173 (33.9)	<0.001
Age, n (%)				
<75 years	151 (26.6)	240 (42.3)	176 (31.0)	
≥75 years	122 (36.3)	100 (29.8)	114 (33.9)	<0.001
Educational level, n (%)				
<Primary	197 (33.5)	193 (32.8)	198 (33.7)	
>Primary	76 (24.1)	147 (46.7)	92 (29.2)	<0.001
Body mass index (kg/m^2^), n (%)				
<25	58 (34.7)	60 (35.9)	49 (29.3)	
25.0–29.9	117 (27.9)	170 (40.6)	132 (31.5)	
≥30	97 (31.2)	110 (35.4)	104 (33.4)	0.40
Waist circumference, n (%)				
Normal	26 (26.8)	42 (43.3)	29 (29.9)	
Moderate	57 (27.9)	95 (46.6)	52 (25.5)	
Large	185 (31.4)	202 (34.2)	203 (34.4)	0.02
Smoking habit, n (%)				
Never	206 (35.9)	176 (30.7)	192 (33.4)	
Current/Past	66 (20.2)	164 (50.2)	97 (29.6)	<0.001
Alcohol consumption, n (%)				
0 g/day	175 (43.4)	88 (21.8)	140 (34.7)	
<12.0 g/day	68 (23.9)	127 (44.7)	89 (31.3)	
≥12.0 g/day	30 (13.9)	125 (57.9)	61 (28.2)	<0.001
Fruit and vegetable intake, n (%)				
<4.3 servings/day	101 (33.6)	109 (36.2)	91 (30.2)	
4.3–6.0 servings/day	91 (30.2)	109 (36.2)	101 (33.6)	
>6.0 servings/day	81 (26.9)	122 (40.5)	98 (32.6)	0.44
Physical Activity at work, n (%)				
Low	104 (35.6)	87 (29.8)	101 (34.6)	
Moderate-high	169 (27.7)	253 (41.4)	189 (30.9)	0.002
Physical activity at Leisure time, n (%)				
Low	153 (30.3)	177 (35.0)	175 (34.7)	
Moderate-high	113 (29.2)	160 (41.3)	114 (29.5)	0.12
Diabetes ^2^ n (%)	50 (28.6)	54 (30.8)	71 (40.6)	0.02
Cholesterol ^2^ n (%)	55 (30.4)	59 (32.6)	67 (37.0)	0.17
Hypertension ^2^ n (%)	120 (33.4)	99 (27.6)	140 (39.0)	<0.001
Sleeping time (hours/day), mean (SD)	7.7 (2.2)	7.9 (1.8)	7.7 (1.9)	0.23
Energy intake (Kcals/day), mean (SD)	1663 (461)	1949 (596)	1800 (541)	0.001

^1^*p*-value from chi-square and ANOVA tests. ^2^ Self-reported diabetes (no/yes), high cholesterol (no/yes) and hypertension (no/yes).

**Table 2:** Associated factors with coffee consumption (caffeinated and decaffeinated) in elderly population, aged 65 years and above, from the EUREYE-Spain Study and the Valencia Nutrition Survey in Spain.

	Caffeinated Coffee		Decaffeinated Coffee	
	RRR ^1^ (95% CI)	*p*	RRR ^1^ (95% CI)	*p*
Sex				
Men	1.00		1.00	
Women	1.37 (0.78–2.41)	0.27	1.27 (0.73–2.21)	0.39
Age				
<75 years	1.00		1.00	
≥75 years	0.64 (0.43–0.94)	0.02	0.83 (0.57–1.21)	0.34
Educational level				
<Primary school	1.00		1.00	
≥Primary school	1.63 (1.09–2.44)	0.01	1.11 (0.73–1.67)	0.63
Body mass index (kg/m^2^)				
Normal (<25)	1.00		1.00	
Overweight (25–29.9)	1.72 (0.99–2.99)	0.05	1.49 (0.84–2.64)	0.17
Obesity (≥30)	2.03 (1.05–3.95)	0.04	1.41 (0.72–2.74)	0.31
Waist circumference (cm)				
Normal	1.00		1.00	
Moderate	0.94 (0.47–1.90)	0.87	0.74 (0.37–1.59)	0.48
Large	0.58 (0.27–1.23)	0.15	0.77 (0.38–1.74)	0.61
Tobacco smoking				
Never	1.00		1.00	
Current/Past	1.96 (1.13–3.39)	0.02	1.45 (0.84–2.51)	0.18
Alcohol consumption				
0.0 g/day	1.00		1.00	
<12.0 g/day	2.86 (1.87–4.36)	<0.001	1.56 (1.03–2.37)	0.04
≥12.0 g/day	6.25 (3.56–10.95)	<0.001	2.63 (1.49–4.64)	0.001
Fruit and vegetable intake				
<4.3 servings/day	1.00		1.00	
4.3–6.0 servings/day	0.88 (0.56–1.38)	0.57	1.03 (0.66–1.60)	0.89
>6.0 servings/day	0.83 (0.51–1.36)	0.46	0.99 (0.61–1.63)	0.98
Physical activity at Leisure time				
Low	1.00		1.00	
Moderate-high	0.78 (0.53–1.15)	0.21	0.74 (0.51–1.09)	0.13
Physical activity at work				
Low	1.00		1.00	
Moderate-high	1.53 (1.00–2.38)	0.06	1.27 (0.84–1.94)	0.26
Diabetes ^2^	1.29 (0.79–2.09)	0.31	1.67 (1.06–2.62)	0.02
Cholesterol ^2^	0.94 (0.58–1.53)	0.81	1.18 (0.76–1.85)	0.46
Hypertension ^2^	0.67 (0.45–0.98)	0.04	1.35 (0.93–1.95)	0.11
Sleeping time (in hours)	1.03 (0.93–1.13)	0.59	0.98 (0.90–1.08)	0.70
Energy intake (x100 kcals)	1.08 (1.03–1.13)	<0.001	1.06 (1.01–1.10)	0.01

^1^ Adjusted relative risk ratios (RRR) from multinomial logistic regression. All variables in the table were included in the model. ^2^ Self-reported diabetes, high cholesterol and hypertension.
